# Chronic musculoskeletal impairment is associated with alterations in brain regions responsible for the production and perception of movement

**DOI:** 10.1113/JP281273

**Published:** 2021-03-23

**Authors:** Veronica Conboy, Carl Edwards, Roberta Ainsworth, Douglas Natusch, Claire Burcham, Buse Danisment, Sharmila Khot, Richard Seymour, Stephanie J. Larcombe, Irene Tracey, James Kolasinski

**Affiliations:** ^1^ Torbay Hospital Torbay and South Devon NHS Trust Newton Rd Torquay TQ2 7AA UK; ^2^ Koç University Hospital Topkapı Koç Üniversitesi Hastanesi Davutpasa Cd. No:4, Zeytinburnu Istanbul 34010 Turkey; ^3^ Cardiff University Brain Research Imaging Centre (CUBRIC) School of Psychology Cardiff University Maindy Road Cardiff CF24 4HQ UK; ^4^ Nuffield Department of Clinical Neurosciences University of Oxford John Radcliffe Hospital Oxford OX3 9DU UK

**Keywords:** brain, cortex, motor, plasticity, rotator cuff

## Abstract

**Key points:**

- Massive irreparable rotator cuff tear was used as a model to study the impact of chronic pain and motor impairment on the motor systems of the human brain using magnetic resonance imaging.
- Patients show markers of lower grey/white matter integrity and lower functional connectivity compared with control participants in regions responsible for movement and the perception of visual movement and body shape.
- An independent cohort of patients showed relative deficits in the perception of visual motion and hand laterality compared with an age‐matched control group.
- These data support the hypothesis that the structure and function of the motor control system differs in patients who have experienced chronic motor impairment.
- This work also raises a new hypothesis, supported by neuroimaging and behaviour, that a loss of motor function could also be associated with off‐target effects, namely a reduced ability to perceive motion and body form.

**Abstract:**

Changes in the way we move can induce changes in the brain, yet we know little of such plasticity in relation to musculoskeletal diseases. Here we use massive irreparable rotator cuff tear as a model to study the impact of chronic motor impairment and pain on the human brain. Cuff tear destabilises the shoulder, impairing upper‐limb function in overhead and load‐bearing tasks. We used neuroimaging and behavioural testing to investigate how brain structure and function differed in cuff tear patients and controls (imaging: 21 patients, age 76.3 ± 7.68; 18 controls, age 74.9 ± 6.59; behaviour: 13 patients, age 75.5 ± 10.2; 11 controls, age 73.4 ± 5.01). We observed lower grey matter density and cortical thickness in cuff tear patients in the postcentral gyrus, inferior parietal lobule, temporal‐parietal junction and the pulvinar – areas implicated in somatosensation, reach/grasp and body form perception. In patients we also observed lower functional connectivity between the motor network and the middle temporal visual cortex (MT), a region involved in visual motion perception. Lower white matter integrity was observed in patients in the inferior fronto‐occipital/longitudinal fasciculi. We investigated the cognitive domains associated with the brain regions identified. Patients exhibited relative impairment in visual body judgements and the perception of biological/global motion. These data support our initial hypothesis that cuff tear is associated with differences in the brain's motor control regions in comparison with unaffected individuals. Moreover, our combination of neuroimaging and behavioural data raises a new hypothesis that chronic motor impairment is associated with an altered perception of visual motion and body form.

## Key points

- Massive irreparable rotator cuff tear was used as a model to study the impact of chronic pain and motor impairment on the motor systems of the human brain using magnetic resonance imaging.
- Patients show markers of lower grey/white matter integrity and lower functional connectivity compared with control participants in regions responsible for movement and the perception of visual movement and body shape.
- An independent cohort of patients showed relative deficits in the perception of visual motion and hand laterality compared with an age‐matched control group.
- These data support the hypothesis that the structure and function of the motor control system differs in patients who have experienced chronic motor impairment.
- This work also raises a new hypothesis, supported by neuroimaging and behaviour, that a loss of motor function could also be associated with off‐target effects, namely a reduced ability to perceive motion and body form.

## Introduction

The structure and function of the brain are shaped by our experiences. This notion has been robustly demonstrated in the human motor system, where changes in the way we move have been associated with alterations in brain activity and brain structure over a period of days or weeks (Acuña *et al*. 1983; Ainsworth, 2006; Ainsworth & Lewis, 2007). The literature on cortical plasticity has focused heavily on studies of training, exercise and drastic insults such as amputation or spinal cord injury (Acuña *et al*. 1983; Andersson *et al*. 2007). However, we know that the implicit changes in the way we move can also cause changes in brain function (Arzy *et al*. 2006; Arrighi *et al*. 2011). Chronic musculoskeletal disease changes the way we move and affects a large proportion of the ageing population, yet there remains a paucity of studies investigating whether these conditions are associated with plastic changes in the motor control systems of the brain (Bach, 1996, 2007; Beckmann *et al*. 2005; Behrens *et al*. 2007), particularly in the upper limb, which engages large and diverse regions of the cerebral cortex (Biagi *et al*. 2010).

Here we use massive irreparable rotator cuff tear as a model disease to investigate whether those suffering from musculoskeletal impairment and pain exhibit differences in brain structure and function compared with unaffected controls. We apply a combination of multimodal magnetic resonance imaging (MRI) and behavioural testing in two separate cohorts of patients and control participants.

Massive rotator cuff tear (hereafter also referred to as cuff tear for brevity) is a condition affecting older adults. Considered in isolation, rotator cuff tear can be regarded as a normal feature of ageing, with tears present in over half of asymptomatic individuals over the age of 60 (Blanke *et al*. 2005). Despite its clinically benign course in many individuals, cuff tear can also be associated with severe pain and profound functional impairment (Born & Bradley, 2005). The rotator cuff is a group of four muscles that functions to stabilise the glenohumeral joint of the shoulder during movement. The combination of pain and shoulder instability in cuff tears can impair affected individuals’ performance of activities involving overhead movement of the arm or lifting a load; dressing, using cutlery, carrying items bimanually, brushing hair, bathing and other everyday domestic tasks can all be affected (Buccino *et al*. 2004). Both tear size and symptoms tend to progress with age (Buxbaum *et al*. 2005; Carter & Huettel, 2013). The precise definition of ‘massive rotator cuff tear’ varies in the literature. Cofield and colleagues (Cleeland & Ryan, 1994) defined it as a tear measuring more than 5 cm in the anterior–posterior or medial–lateral dimensions. Gerber and colleagues (Cofield, 1982) defined it as involving complete tears of at least two tendons. A recent consensus study arrived at a definition of more than two‐thirds of the attachment exposed, with retraction of tendon(s) to the glenoid rim (Coombes *et al*. 2011). The management of massive tears is challenging in some cases, not least because structural failure does not always equate to clinical failure (Cofield, 1982), and the outcome of repair or attempted repair may be poor (Dale *et al*. 1999). Non‐operative management in cases of massive rotator cuff tear may be indicated when the tear is longstanding and associated with severe functional deficit, muscle belly degeneration (fatty infiltration), and/or secondary glenohumeral arthropathy (Dawson *et al*. 2009; Deen & McCarthy, 2010). It usually takes the form of rehabilitative physiotherapy (Draganski *et al*. 2004; Douaud *et al*. 2007). The chronic nature of massive irreparable rotator cuff tear makes it an appealing model disease through which to study the motor systems of the brain focusing on the question: how do the motor systems of patients who have experienced long‐term motor impairment and pain differ from the brains of unaffected individuals of the same age?

In Study A, we used a cross‐sectional design to investigate whether chronic massive rotator cuff tear is associated with differences in brain structure and function compared with a group of unaffected control participants. We used MRI to assess the structural integrity of the brain's grey matter and white matter, cortical thickness, and measures of functional connectivity from resting‐state functional MRI (fMRI). We hypothesised that musculoskeletal impairment in the upper limb will be associated with lower structural integrity in motor control regions and their underlying white matter connections. Furthermore, we expected to observe evidence of localised differences in the connectivity of the brain's motor network, measured using resting‐state fMRI.

In Study B, we used these brain imaging data to guide an investigation of the behavioural impairments associated with massive rotator cuff tear. We recruited an independent cohort of cuff tear patients and control participants to investigate how the brain differences observed in Study A might manifest in terms of function. The behavioural measures under study were dictated by the results of Study A. By recruiting an independent cohort in each study, we avoided the potential pitfalls of circular inference.

## Methods

### Ethical approval

All participants were recruited from Torbay Hospital in accordance with local NHS Research Ethics Committee approval (South‐West NRES REC: 10/HO102/35) and the standards set out by the *Declaration of Helsinki* (2013). All participants gave written informed consent to take part in the study.

### Study A: neuroimaging

#### Participants

A total of 21 patients with massive irreparable rotator cuff tear were recruited from the orthopaedic outpatient clinic at Torbay Hospital (mean age 76.3 ± 7.68; three female; three left‐handed; 11 were to be treated for a tear in the right shoulder; six had bilateral tears). An irreparable tear was classified as a large or massive tear (5 cm or longer), significantly retracted, involving at least the whole of the attachment of supraspinatus to the humeral head, possibly also involving the infraspinatus and subscapularis attachments, with a history suggestive of a longstanding problem rather than a single acute traumatic event. In association, patients exhibited significant local muscle wasting and plain radiographic changes suggestive of longstanding derangement (Coombes *et al*. 2011).

All patients were older than 18 years of age, with a diagnosis of a full thickness tear of over 5 cm confirmed by surface ultrasound. Exclusion criteria were as follows: the presence of any neurological condition; contraindication for MRI; involvement of the individual in an industrial claim or litigation related to shoulder injury; history of recent shoulder trauma; or the presence of a rotator cuff tear considered to be repairable by a consultant orthopaedic surgeon. A total of 18 control participants unaffected by massive irreparable rotator cuff tear were also recruited (mean age 74.9 ± 6.59; six female). Exclusion criteria included the presence of any neurological condition, contraindication for MRI, and the presence of any shoulder pathology or rotator cuff tear.

All control participants were screened for the presence of shoulder pathology with a clinical examination and surface ultrasound.

#### Patient and public involvement

Neither a patient group nor the public were involved in the design, undertaking, reporting, or dissemination plans involved in this study.

#### Experimental design

All patients and control participants attended an initial screening session, followed by an MRI scan session. The screening consisted of the collection of baseline clinical scores and a clinical examination of the shoulder, including an ultrasound to confirm or rule out the presence of rotator cuff tear.

#### Sample characteristics and clinical measures

Age, sex and handedness were recorded for all participants during their initial screening session. The Oxford Shoulder Score (OSS) was used to quantify shoulder function in all participants (Oxford University Innovation; Oxford, UK) (Ferrari *et al*. 2017). This is a well‐validated 12‐item, patient‐reported, questionnaire‐based metric used commonly to assess surgical outcomes, graded from 0 (the most severe symptoms) to 48 (normal function). In addition, the brief pain inventory short form (BPI‐sf) was used to assess pain intensity by asking participants to rate their pain across four scales from 1 to 10 from ‘no pain’ to ‘pain as bad as you can imagine’ describing: (a) the most pain experienced in the last 24 h, (b) the least pain experienced in the last 24 h, (c) the average level of pain, and (d) pain right now (Filippini *et al*. 2009).

#### MRI data acquisition

MRI data were acquired at Torbay Hospital using a General Electric 1.5 T Signa HD system (General Electric, Fairfield, CT, USA) with an eight‐channel head coil and maximum 23 mT m^−1^ gradient capability. Structural MRI data were acquired using a T1‐weighted 3D inversion recovery prepared *FSPGR* sequence (matrix 256 × 265, 1.0 × 1.0 × 1.0 mm resolution, 170 axial slices; echo time (TE), 3.76 ms; repetition time (TR), 9.3 ms; flip angle, 13°). Diffusion‐weighted MRI data (dw‐MRI) were acquired using a spin echo echo‐planar imaging (EPI) sequence (matrix 256 × 256, 0.94 × 0.94 × 2.5 mm resolution, 50 axial slices; TE, 93.6 ms; TR, 13000 ms; bandwidth, 1953 Hz; flip angle, 90°) with 60 isotropically distributed diffusion directions (*b*‐value: 1000 s mm^−2^) and six *b* = 0 images. Resting state fMRI data were acquired using a 2D gradient echo EPI sequence (matrix 128 × 128, 2 × 2 × 5 mm resolution, 28 axial slices, 125 measurements; TE, 60 ms; TR, 3000 ms; bandwidth, 3906 Hz; flip angle, 90°).

#### MRI analysis

All MRI analysis was undertaken using the FMRIB Software Library (FSL) (Fiorio, 2005). The anatomical localisation of statistical maps generated from MRI data was guided by the Glasser cortical atlas (Fischl *et al*. 2001). Functional definitions of the anatomical hand knob were provided by the O'Neill probabilistic atlas (Fogassi & Luppino, 2005).

##### Voxel‐based morphometry

T1‐weighted MRI data were used in voxel‐based morphometry (VBM) analysis. T1‐weighted data were first corrected for bias field inhomogeneity using fsl_anat. VBM analysis was then conducted using FSL‐VBM (Gerber *et al*. 2000), an optimised VBM protocol (Glasser *et al*. 2016). First, structural images were brain‐extracted and grey matter‐segmented before being registered to the MNI152 standard space using non‐linear registration (Good *et al*. 2001). The resulting images were averaged and flipped along the *x*‐axis to create a left‐right symmetric, study‐specific grey matter template from an equal number of participants in the patient and control groups. Second, all native grey matter images were non‐linearly registered to this study‐specific template and ‘modulated’ to correct for local expansion (or contraction) due to the non‐linear component of the spatial transformation. The modulated grey matter images were then smoothed with an isotropic Gaussian kernel with a sigma of 3 mm. Finally, voxelwise general linear modelling (GLM) was applied using permutation‐based non‐parametric testing, correcting for multiple comparisons across space.

##### FreeSurfer cortical thickness analysis

Cortical surface reconstruction was undertaken using T1‐weighted MRI data in FreeSurfer Version 7.1.0 (Grieve *et al*. 2000; Greve & Fischl, 2009). Whole‐brain vertex‐wise comparisons of cortical thickness were computed using a GLM contrasting the patient and control groups with cluster‐wise correction for multiple comparisons (1000 permutations; vertexwise threshold *P* < 0.001; cluster‐wise threshold *P* < 0.05).

##### fMRI resting‐state network analysis

fMRI data were subject to standard preprocessing, including motion correction using MCFLIRT (Griffanti *et al*. 2017), brain extraction using BET (Gwilym *et al*. 2010), and high‐pass temporal filtering (100 s threshold). fMRI data were subject to spatial smoothing using an isotropic Gaussian kernel with a sigma of 5 mm. All fMRI data were subject to manual independent components analysis (ICA) denoising prior to further analysis (Hétu *et al*. 2013). fMRI data were aligned with T1‐weighted images using FMRIB's linear image registration tool (FLIRT) optimised with boundary‐based registration (Howard *et al*. 2019). Non‐linear transformations to MNI152 standard space were used to transform fMRI data into a common space. Data across all participants in both groups were concatenated to create a single 4D data set. This data set was subject to a dual regression analysis implemented in FSL (Husain & Nachev, 2007), which can be described in three steps. Equations and description thereof are adapted from Nickerson *et al*. (2017) and Iacoboni & Dapretto (2006).

Initially, the concatenated 4D fMRI data set was subject to a group ICA (25 components limit). In a second step, the resting state fMRI data for each participant (*Y*) is reshaped into a data matrix (*N* voxels × *T* time points). For each participant the group‐average template resting‐state networks were regressed into this data matrix (as a spatial regressor in a multiple regression), where $\hat{S}$ is the resting‐state template maps reshaped into a 2D matrix (*N* voxels, *M* components); these maps for each component ($\hat{S}$) form the predictors for a multivariate multiple linear regression:

(1) $$Y=\hat{S}B_{\mathrm{TC}}+E_{1}$$

with

(2) $${\hat{B}}_{\mathrm{TC}}=\mathrm{pinv}\left(\hat{S}\right)Y$$

pinv refers to the matrix pseudoinverse, $Y\;\in R^{N\times T}$ is the resting state fMRI data of an individual participant, $E_{1}\;\in \;R^{N\times T}$ is the matrix of errors, and ${\hat{B}}_{\mathrm{TC}}\in R^{N\times T}$ is the matrix of time courses for each resting‐state template map for the participant in question: each time course represents the average across each resting‐state template map accounting for the contributions of other resting‐state map time series to each voxel's time course. The time series that make up $\hat{S}$ are the same across all participants.

In a third step, the resting‐state network specific time series from the regression conducted in Stage 2 were used as predictors in a further multivariate multiple linear regression of each participant's 4D fMRI data set. This second regression resulted in a spatial map for each of the template resting state networks for each participant, *B*_SM_

(3) $$Y^{\prime }={\hat{B}}_{\mathrm{TC}}^{\prime }B_{\mathrm{SM}}+E_{2}$$

Which gives:

(4) $${\hat{B}}_{\mathrm{SM}}=Y\times \mathrm{pinv}\left({\hat{B}}_{\mathrm{TC}}\right)$$

The estimated ${\hat{B}}_{\mathrm{SM}}\in R^{N\times T}$ contains a spatial map for each of the resting‐state templates from the original group ICA; these are the maps of resting‐state functional connectivity for each network. The resting‐state motor network of interest was identified using spatial correlations against reference data (Ilg & Schumann, 2007).

The resting‐state motor network maps were compared across the patient and control groups using voxelwise GLM with permutation‐based non‐parametric testing, correcting for multiple comparisons across space. In addition, the global strength of the motor network was assessed across the two groups by masking the subject‐specific resting‐state motor network maps from each participant's data with a mask derived of the group mean resting‐state motor network. This process yielded a measure of functional connectivity strength within the motor network.

##### White matter tract‐based spatial statistics

dw‐MRI data were initially corrected for head‐motion and eddy‐current distortions using the FMRIB Diffusion Toolkit (Ionta *et al*. 2011). Voxelwise analysis of dw‐MRI was conducted using tract‐based spatial statistics (TBSS) (Jaywant *et al*. 2016). Fractional anisotropy (FA) images were calculated by fitting a diffusion tensor model using DTIFIT. Data were brain extracted and aligned to a common space using FNIRT. Next, the mean FA image was created and thinned to generate a mean FA skeleton which represents the centres of all tracts common to the group. Each participant's aligned FA data were then projected onto this skeleton and the resulting data were fed into a voxelwise GLM using permutation‐based non‐parametric testing, correcting for multiple comparisons across space.

##### Statistical comparison of neuroimaging data across groups

To examine differences between the patients with cuff tear and the unaffected control participants, VBM, fMRI dual regression and TBSS analyses compared neuroimaging metrics across groups using voxelwise GLMs. Results within each modality were corrected for multiple comparisons using threshold‐free cluster enhancement (TFCE) (5000 permutations; *P*_FWE_ < 0.05; FWE, family wise error) via non‐parametric testing in FSL Randomise (Jenkinson *et al*. 2012). All statistical neuroimaging analysis included sex, age, dominant hand and the presence of a bilateral cuff tear as covariates.

Correction for comparison across multiple imaging modalities was based on clear directional hypotheses in each analysis (VBM: reduced grey matter density in patients; dw‐MRI: reduced FA in patients; rs‐fMRI: reduced motor network connectivity in patients). This correction used a conjunction test across all modalities. In this stringent analysis, the compound null hypothesis can only be disproved if all of the predicted group differences across modalities are significant. The conjunction test therefore considered the maximum (if any) significant corrected *P*‐value across predicted group comparisons.

##### Meta‐analytic assessment of neuroimaging results

The observed spatial maps demonstrating group differences were further contextualised using Neurosynth, a sophisticated MRI meta‐analysis tool available via a web platform for large‐scale, automated analyses of cognitive domains associated with statistical maps generated by brain imaging (Jenkinson *et al*. 2002). Neurosynth compared the MNI coordinates of significant clusters from the VBM and dual regression analyses to functional activation maps measured from over 14,300 reference fMRI activation maps reported in the primary literature; each reference map is associated with specific cognitive domains. The result of this analysis was a statistical association between specific cognitive domains and the brain regions highlighted by our VBM and dual regression analyses. Each of these cognitive domains is represented by a keyword and scaled by the associated *Z*‐score, which represents the strength of the association between the specific cognitive domain and the brain region highlighted by our statistical analysis. The aim of this approach is to avoid selectively associating significant results in specific brain regions with specific cognitive domains, as this tool looks indiscriminately across over 14,300 published studies. A full description of the calculation of these *Z*‐scores using an association test with specific MNI coordinates is provided here.

### Study B: behavioural testing

#### Participants

On the basis of the results reported in Study A, an additional naive cohort of patients with cuff tear and an unaffected control group were recruited into a behavioural study assessing visual perception of motion and body form. Using the same recruitment methods, inclusion/exclusion criteria (except those pertinent to MRI), and screening described above, 13 patients with cuff tears (mean age 75.5 ± 10.2; five female; all right‐handed; 11 were to be treated for a tear in right shoulder; three had bilateral tears) and 12 unaffected controls (mean age 73.4 ± 5.01; five female) were recruited.

#### Experimental design

All patients and control participants attended an initial screening session, as outlined above, followed by a behavioural testing session.

#### Measures of visual acuity and static contrast sensitivity

Measures of visual acuity and static contrast sensitivity were collected using the Freiburg Visual Acuity and Contrast Tests (Johansen‐Berg *et al*. 2005; Juhan *et al*. 2019). Both acuity (decimal acuity: VA_DEC_) and contrast sensitivity (Weber contrast: logCS_Weber_) were measured using a Landolt C with four potential orientation choices, at a distance of 4 m from the screen (24 trials each).

#### Motion stimuli

All visual motion stimuli were programmed using MATLAB (vR2012a, The MathWorks, Natick, MA, USA) with Psychtoolbox (v3.0, http://psychtoolbox.org), and were presented on a 54cm monitor (Apple iMac, 1920 × 1080 pixel resolution, 60 Hz refresh rate) in a darkened room. Participants were positioned approximately 57 cm from the screen. At the beginning of each task, participants undertook an initial 10 practice trials to familiarise themselves with the task. During the practice trials, participants received visual feedback via the fixation cross after each trial (green: correct; red: incorrect). During all trials in the main task, a central fixation cross was present but remained white throughout; participants were instructed to focus on the fixation cross at all times. In all motion tasks, an easy ‘catch’ trial was included randomly within each of the 20 trials. In the global motion task, these ‘catch’ trials involved 100% coherence stimuli. In the walker task, the trials involved a walking figure without any overlaid moving dot field. The purpose of these relatively easy trials was to provide a metric of attention to exclude participants who were responding without engaging with the task. Participants who performed <75% on catch trials were excluded.

##### Global visual motion perception

Participants were asked to judge whether a field of moving dots presented against a black background had either a leftward or a rightward coherent overall motion. The direction of coherent motion was always restricted to within a 90° angle centred on the horizontal axis. Moving dots (*n* = 143) were presented within a circular window 11° in diameter, offset 10° to the left or right of fixation (dot diameter: 0.15°; dot speed: 4°/s; dot lifetime: 200 ms; dot density: 3.0 dots/degree^2^; duration: 500 ms). Dots were regenerated within the circular window in random positions not overlapping with existing dots. Perception in the left and right hemifields was tested separately in two task blocks, each comprising 60 trials, with a total of 120 trials overall.

In each trial, participants were presented with a moving dot stimulus, followed by a response window (see Fig. 6). Participants indicated which direction the stimulus was moving in using the left and right arrow keys on a keyboard. Their response triggered the start of a new trial. An optional screen break was offered every 20 trials to minimise fatigue.

##### Measure of biological visual motion perception

Participants were asked to judge whether a point‐light motion profile (22 dot walker; Kairys *et al*. 2015; Fig. 6) was walking forwards or backwards on the spot. The two stimuli were generated from identical recordings, one of which had been reversed. The point‐light walker was overlaid with a field of moving dots (121 dots) with the same characteristics outlined in the global motion task. During each trial, participants were instructed to focus on the fixation cross while a 1000 ms walker stimulus was presented, followed by a response window. Participants indicated which direction the walker was moving in using the left and right arrow keys on a keyboard, triggering the start of the next trial. Participants were initially presented with 100 trials. As in the global motion task, an optional break was provided every 20 trials. In each trial, the circular window in which the walker and background dots were presented was spatially shifted ±2° in the *x* and *y* axes to prevent the use of strategies involving fixation on a single walker dot. The starting frame of the walker was randomly selected in each trial.

##### Analysis of motion stimuli

In both the global and biological motion tasks, dot coherence (excluding the walker dots) was adaptively modulated in two interleaved two‐down/one‐up staircases to adjust difficulty. In order to more quickly reach a coherence threshold, the step size prior to the first incorrect response was set to a fractional decrement of 0.5 in each staircase. Thereafter, the fractional decrement/increment associated with correct or incorrect answers was 0.75/1.25 respectively. Motion direction discrimination thresholds were calculated as the mean of the coherence at the final 12 staircase reversals (where difficulty shifted from increasing to decreasing or vice versa).

#### Measures of visual body perception

##### Hand laterality task

The hand laterality task was closely matched to that described previously by Maimon and colleagues (Kim *et al*. 2018). Participants were presented with egocentric photographs of the right hand in 24 different positions, ranging from simple to complex. These images were digitally mirrored to create identical images of a left hand in the same position. In a single experimental block, participants were presented with each of these images in a random order using PsychoPy (Version 1.84.20) (Kriegeskorte *et al*. 2009; Kolasinski *et al*. 2016). Participants were asked to judge the laterality of each image (i.e. whether it contained a left or right hand) through a response on a gamepad. Importantly, participants’ view of their own hands was physically obstructed by a desk, and participants were instructed to keep their hands in a fixed position while gripping the gamepad during the task. Participants were also instructed to respond as quickly and accurately as possible. Each image was preceded by a 1‐s fixation cross. Both their reaction time and their response were recorded for each trial. The participant's response triggered the beginning of the next trial.

##### Analysis of laterality reaction time and accuracy

Accuracy was defined as the proportion of correct trials out of all trials for which a response was recorded. Reaction times were quantified as an average across all correct trials.

#### Statistical analysis of behavioural measures

To test the hypothesis that massive irreparable rotator cuff tear is associated with an altered perception of motion and body form but not low levels of visual perception of contrast or acuity, we used distinct statistical tests to detect any predicted and unpredicted differences between the patient and control groups. Specifically, we used a conjunction test over all comparisons predicted to be significant and an omnibus test over all comparisons predicted to be non‐significant (Lanzilotto *et al*. 2019). The conjunction test considered the maximum *P*‐value across group comparisons that were predicted to be significant. In this stringent analysis, the compound null hypothesis can only be disproved if all of the predicted comparisons of global motion perception, biological motion perception and body perception are significant. The omnibus test considered the minimum *P*‐value across all group comparisons predicted to be non‐significant, that is, contrast acuity and visual acuity. While one can never prove the null hypothesis, this liberal test should enable the detection of any possible group differences in low‐level perceptual abilities across the two groups.

## Results

### Motor impairment and pain in cuff tear patients

**Figure 1:**
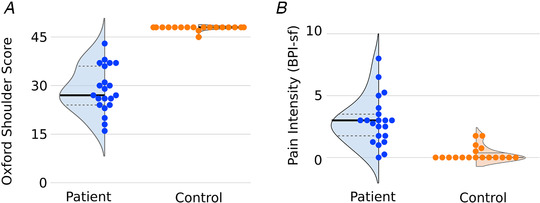
Patients with massive irreparable rotator cuff tear show reduced shoulder function (*A*, lower OSS) and higher levels of pain intensity (*B*, BPI‐sf) than unaffected control participants Data shown for Study A cohort; equivalent values for Study B are reported in the main text. Median indicated by bold lines; quartiles indicated by dashed lines. [Color figure can be viewed at wileyonlinelibrary.com]

As predicted, in both cohorts, cuff tear patients showed lower shoulder function and higher pain scores than unaffected control participants (Fig. 1).

In Study A, the OSS was significantly lower in patients (mean (SD) 28.73 (6.96)) than controls (47.78 (0.73); *t*(21.6) = 12.7, *P* = 0.001, Cohen's *d* = 3.66). Pain intensity measured using the BPI‐sf was significantly higher in patients (3.03 (1.92)) than controls (0.393 (0.645); *t*(27.3) = 6.22, *P* = 0.001, Cohen's *d* = 1.81).

In Study B, the OSS was also significantly lower in patients (23.46 (7.73)) than controls (47.5 (1.73); *t*(13.3) = 10.9, *P* = 0.001, Cohen's *d* = 4.20). Pain intensity measured using the BPI‐sf was significantly higher in patients (2.15 (1.58)) than controls (0.437 (0.545); *t*(15.0) = 3.69, *P* = 0.002, Cohen's *d* = 1.42).

In Study A, one patient was excluded from all analyses due to an incidental finding: an undiagnosed pathology was identified during MRI. In Study B, one patient and one control participant were excluded due to a failure to complete the behavioural tasks (performed with less than 75% accuracy on catch trials).

### Massive irreparable rotator cuff tear patients show lower grey matter density in primary somatosensory cortex, anterior intra‐parietal sulcus, inferior parietal lobule and temporal–parietal junction (Study A)

**Figure 2:**
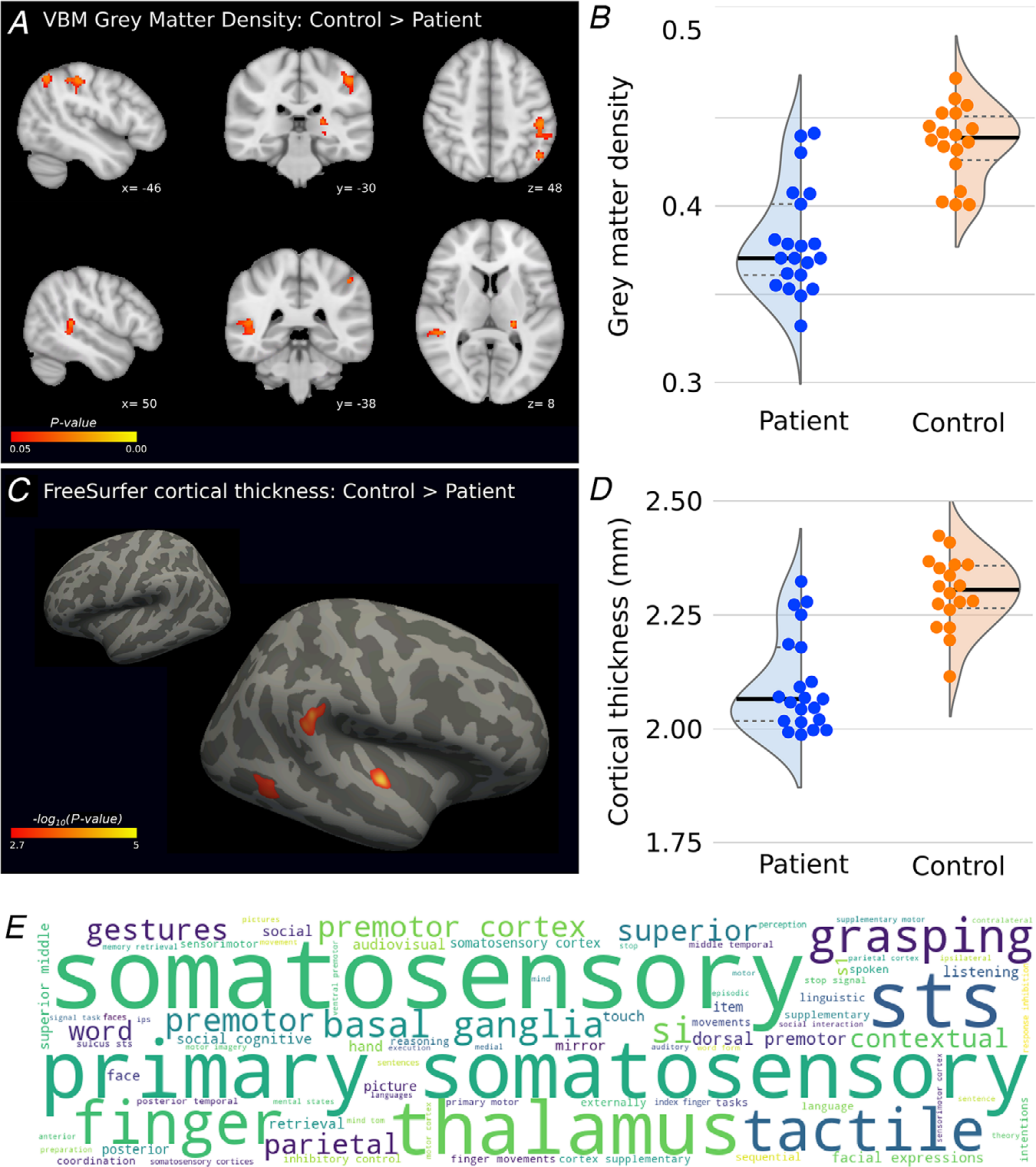
Evidence of localised cortical regions of lower cortical grey matter density and cortical thickness in cuff tear patients *A* and *B*, massive irreparable rotator cuff tear patients have lower grey matter density in left primary somatosensory cortex, left inferior parietal lobule, right temporal–parietal junction, and left thalamus (posterior parietal projection) (*P*_FWE_ < 0.05, TFCE). See Table 1 for all cluster locations. *C* and *D*, cuff tear patients also show lower cortical thickness in right temporal–parietal junction, right superior temporal sulcus, and right inferior temporal cortex (vertex‐wise threshold *P* < 0.001; cluster‐wise threshold *P* < 0.05). *E*, neurosynth keyword meta‐analysis shows cognitive functions and brain areas co‐localised to brain regions identified in *A* from 14,300 reference fMRI studies; word size ≈ strength of association with the spatial map scaled by *Z*‐score (max: 8.9; min: 3.6). [Color figure can be viewed at wileyonlinelibrary.com]

Grey matter VBM analysis revealed significantly lower grey matter density in the cuff tear group compared with unaffected controls (Fig. 2 *A* and *B*). These differences were observed in the left postcentral gyrus (S1; Glasser atlas: area 2); left anterior intra‐parietal sulcus (AIP; Glasser atlas: area PF complex); left inferior parietal lobule (Glasser atlas: area PFm complex); right temporal‐parietal junction around the superior temporal sulcus (Glasser atlas: temporo‐parieto‐occipital junction [TPOJ1] and superior temporal sulcus‐dorsal posterior [STSdp]); and left thalamus (posterior parietal projection; pulvinar nucleus) (Leube *et al*. 2003; Maeda *et al*. 2016) (Table 1). No significant regions of lower grey matter density were observed in unaffected control participants relative to cuff tear patients.

**Table 1:** Overview of significant VBM clusters

Cluster location	Voxels	Max *X*	Max *Y*	Max *Z*
Left postcentral gyrus/AIP	177	−48	−28	46
Left inferior parietal lobule	166	−56	−64	32
Right anterior TPJ	145	48	−36	10
Left thalamus (PPP; pulvinar)	65	−22	−28	8
Right posterior fusiform	44	36	−38	−20
Right posterior TPJ/STS	11	56	−50	32

Regions where grey matter density is greater in controls than cuff tear patients; all coordinates in MNI standard space (mm). AIP, anterior intra‐parietal sulcus; PPP, posterior parietal projection; STS, superior temporal sulcus; TPJ, temporal‐parietal junction.

In a further analysis using FreeSurfer cortical reconstructions, cuff tear patients also showed lower cortical thickness in the right temporal–parietal junction (TPJ) and superior temporal sulcus, regions complementary to the reduced grey matter density revealed by VBM. Additional areas of lower cortical thickness were observed in the right inferior temporal cortex (vertex‐wise threshold, *P* < 0.001; cluster‐wise threshold, *P* < 0.05) (Fig. 2 *C* and *D*).

Regions of reduced grey matter density in the patient group were further interrogated using the Neurosynth fMRI meta‐analysis database. This analysis showed a clear association between the regions of reduced grey matter density in patients and a broad range of somatosensory and motor functions pertaining to the upper limb (Fig. 2 *E*).

### Massive irreparable rotator cuff tear patients show altered motor network connectivity with middle temporal visual cortex (MT) involved in motion and action perception (Study A)

**Figure 3:**
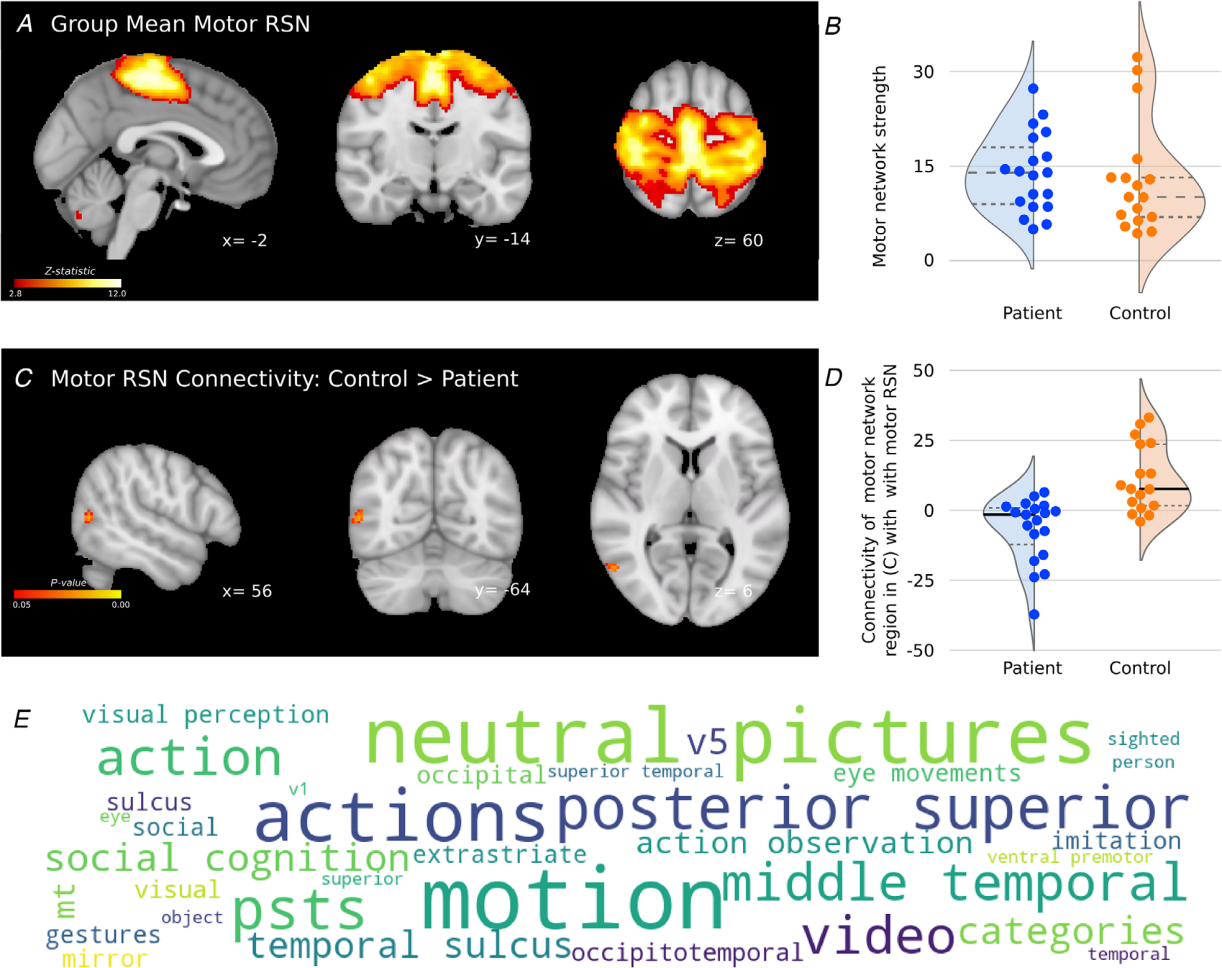
Massive irreparable rotator cuff tear patients show reduced functional connectivity between the motor network and MT cortex, which is implicated in motion and action perception *A*, group mean resting‐state motor network. *B*, group comparison of the strength of motor network connectivity reveals no global difference between groups. *C*, a dual regression analysis identified a region of MT as a region of differing functional connectivity with the resting‐state motor network between cuff tear patients and control participants. *D*, *post hoc* visualisation of the functional connectivity measures in the region of MT identified. *E*, keyword meta‐analysis using Neurosynth to quantify the strength of associations between specific cognitive functions and the brain regions identified in *C* from ∼14,300 reference fMRI studies. Word size represents relative strength of association with spatial map (*Z*‐score: max, 9.88; min, 3.48). RSN, resting‐state network. [Color figure can be viewed at wileyonlinelibrary.com]

The resting‐state motor network was identified from a 25‐component ICA and subject to a dual regression analysis (Fig. 3 *A–D*). Comparison of the resting‐state motor networks across cuff tear patients and control participants revealed a difference in resting‐state functional connectivity localised to middle temporal visual cortex (MT) (Glasser atlas: MT/TPOJ2). Plotting connectivity values from the dual regression analysis within this region revealed clear dissociation in connectivity between cuff tear patients and unaffected control participants. This region of MT showed a positive correlation with the activity of the resting‐state motor network in the control group. In contrast, the patient group showed a negative correlation between the pattern of activity in MT and the resting‐state motor network. In other words, the spontaneous activity of MT is more closely synchronised to the motor network in unaffected controls than in cuff tear patients, where MT activity is weakly correlated or anticorrelated to the resting‐state motor network. The overall strength or intrinsic functional connectivity of the motor network did not differ between patients and control participants (*t*(34) = 0.387, *P* = 0.701; Fig. 3 *B*).

Further interrogation of this region of MT cortex using the Neurosynth fMRI meta‐analysis database revealed a clear functional relevance of this region to motion perception and action observation (Fig. 3 *E*).

### Massive irreparable rotator cuff tear patients show lower white matter FA in inferior frontal occipital fasciculus/inferior longitudinal fasciculus underlying regions of structural and functional differences (Study A)

**Figure 4:**
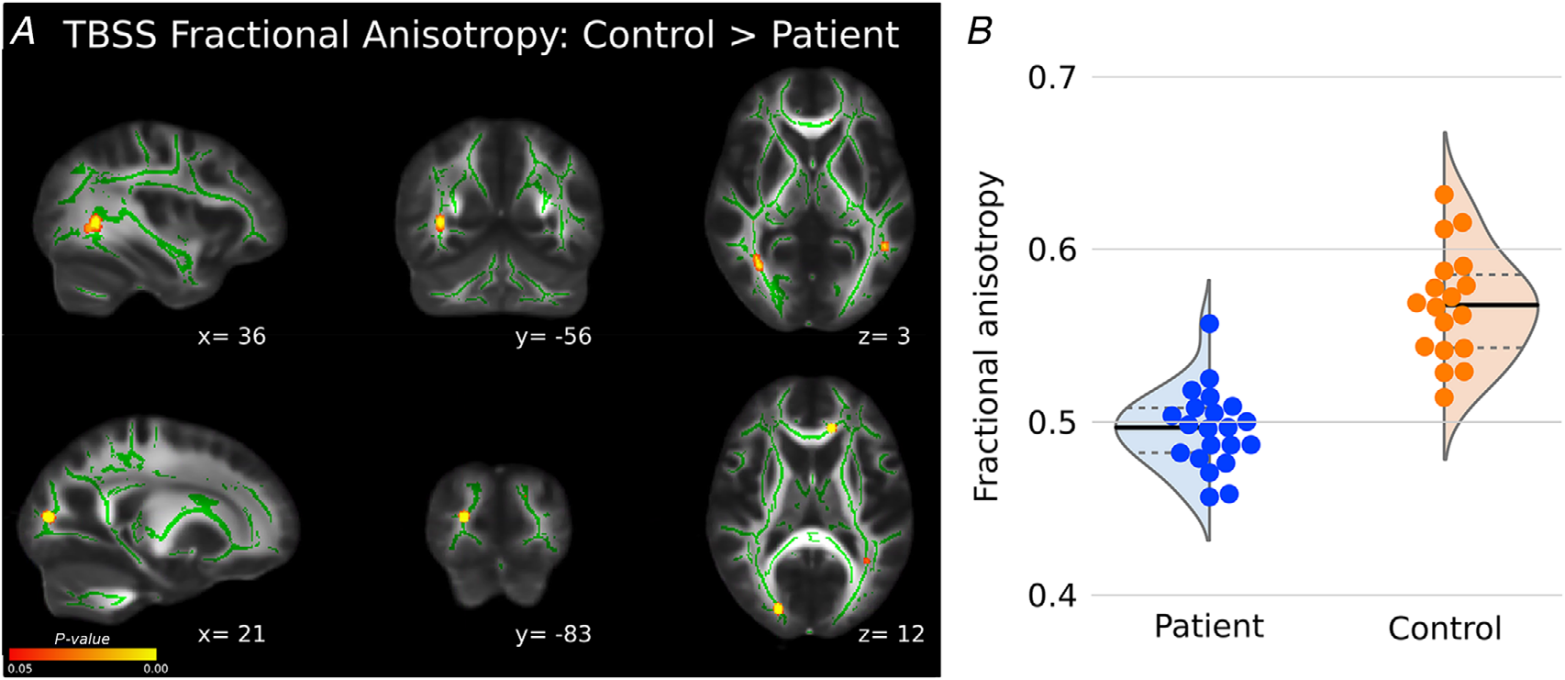
TBSS reveal evidence of reduced white matter integrity in localised regions including right inferior frontal occipital fasciculus and inferior longitudinal fasciculus *A*, cuff tear patients showed lower FA in localised white matter regions including right inferior frontal‐occipital fasciculus, inferior longitudinal fasciculus and optic radiations, including areas directly adjacent to grey matter and functional connectivity differences (Figs 2 and 3). *B*, *post hoc* visualisation of FA values in significant regions across groups. [Color figure can be viewed at wileyonlinelibrary.com]

**Figure 5:**
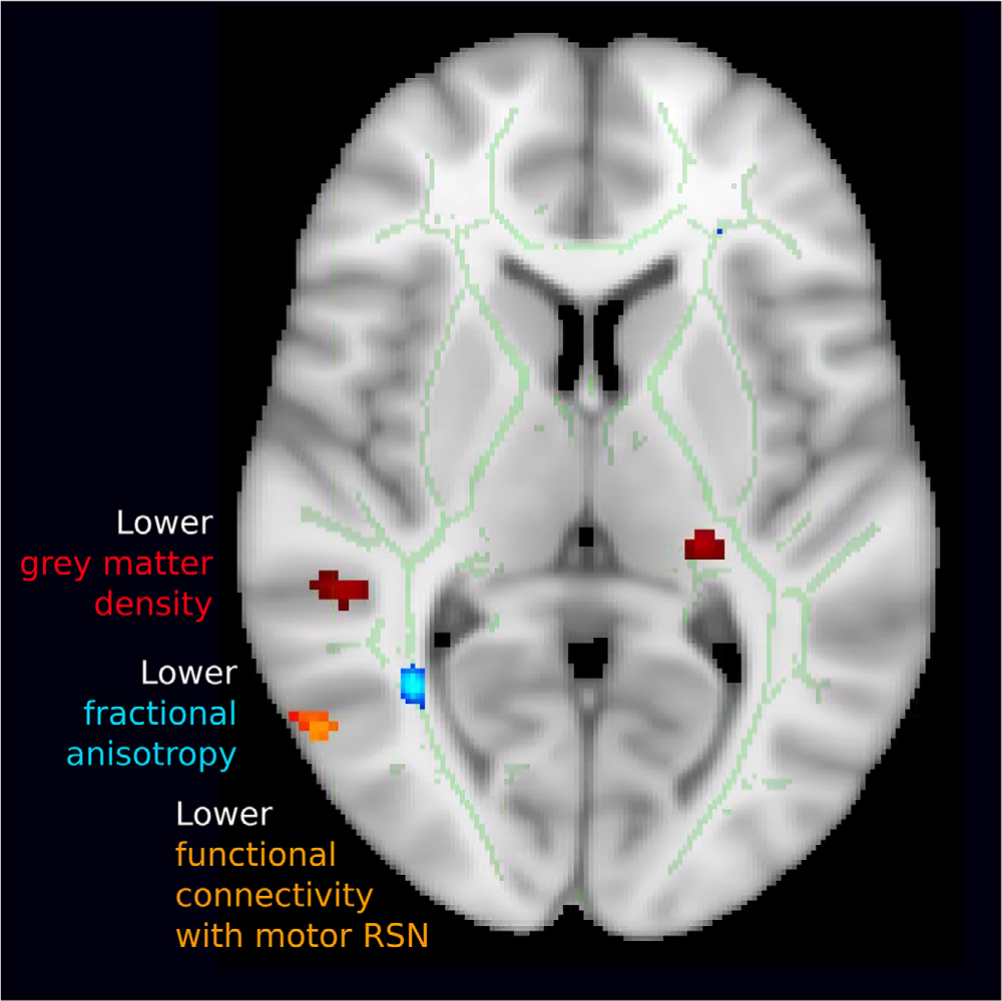
Summary of multimodal structural and functional differences between cuff tear patients and controls co‐localised to a cortical region associated with motion and action observation Observed differences in grey matter density (red), white matter integrity (blue) and functional connectivity of the motor network (orange), derived from three entirely independent modalities and analyses, are co‐localised to a region responsible for body perception and motion. [Color figure can be viewed at wileyonlinelibrary.com]

Localised reductions in white matter FA were observed in the TBSS white matter skeleton in the left and right inferior frontal occipital fasciculus (IFOF) and inferior longitudinal fasciculus (ILF) (Fig. 4 *A/B*). Importantly, this observed cluster of lower white matter integrity was directly adjacent to the observed clusters of reduced functional connectivity in right MT cortex and reduced grey matter density in right anterior TPJ (TPJa) in cuff tear patients (Fig. 5). Further regions of lower FA in cuff tear patients were also observed in right superior longitudinal fasciculus and right optic radiations.

### Massive irreparable rotator cuff tear patients show poorer perception of visual motion and body form compared with control participants (Study B)

The results of Study A revealed not only reduced structural integrity in regions associated with sensorimotor function (e.g. primary somatosensory cortex) but also multimodal evidence for impaired structure and function in higher order visual regions pertaining to motion perception and action observation. An independent cohort of cuff tear patients and unaffected control participants was therefore recruited for a behavioural study (Study B) to assess whether the evidence of structural and functional deficits in higher order visual regions from the MRI was mirrored in visual function. Specifically, we used tests of global and biological motion perception further investigating the imaging results observed in MT, and tests of body form to further explore the observed grey matter differences in TPJ and the underlying white matter.

There was no significant evidence for a difference between Study B cuff tear patients and the control group in terms of visual acuity (VA_DEC_) or contrast sensitivity (Weber contrast: logCS_Weber_) (multivariate analysis of variance (MANOVA) *F*(2,19) = 1.54, *P* = 0.239; Wilk's lambda = 0.0833; *post hoc t*‐test minimum value *P* = 0.161).

There was evidence of statistically significant differences in the perception of visual motion (global and biological stimuli) and body form (hand laterality stimuli) across the cuff tear and control groups (MANOVA *F*(3,19) = 3.454, *P* = 0.0371; Wilk's lambda = 0.647). Follow‐up tests of these pairwise relationships used Welch's independent samples *t*‐test (Maimon‐Mor *et al*. 2020). In the global motion perception task, the mean coherence threshold for cuff tear patients (0.355 (0.143)) was significantly higher than for the control group (0.223 (0.102); *t*(19.9) = 2.56, *P* = 0.009, Cohen's *d* = 1.05). In the biological motion task, the mean coherence threshold for cuff tear patients (0.864 (0.129)) was also significantly higher than for the control group (0.676 (0.308); *t*(13.1) = 1.88, *P* = 0.041, Cohen's *d* = 0.81).

This observation shows that control participants were significantly better at discriminating global and biological motion stimuli from noise stimuli than cuff tear patients. In the body perception task, the reaction time of cuff tear patients on correct trials (2.15 (1.16) s) was significantly longer than those of the control group (1.35 (0.58) s); *t*(16.5) = 2.12, *P* = 0.025, Cohen's *d* = 0.861). This observation shows that cuff tear patients were slower to make a correct judgement of hand laterality from a range of images involving different hand positions.

A conjunction test supported the hypothesis, based on the brain imaging results in Study A, that cuff tear patients would show poorer performance of body perception and visual motion tasks (maximum *P*‐value of predicted group differences: *P* = 0.041). In contrast, an omnibus test across the measures of visual motion where no group differences were expected (visual acuity and contrast acuity) revealed no evidence of any significant group differences (minimum *P*‐value of unpredicted group differences: *P* = 0.161). This combination of an omnibus and a conjunction test allows us to confirm our hypothesis that cuff tear patients show deficits in higher order perceptual functions but not in lower level visual/contrast acuity measures.

Further analysis of the biological and global motion perception task data revealed no difference between the patient and control groups in terms of reaction time (mixed ANOVA; between subjects factor: group; within subjects factor: visual motion task – biological or global motion; main effect of group *F*(1,23) = 0.326, *P* = 0.574, η_p_^2^ = 0.014). These tasks used stimuli of fixed duration and prompted a response from participants with a visual cue.

They therefore provide a valuable measure of reaction time independent of any concurrent visual or cognitive load.

## Discussion

We present clear evidence from neuroimaging and behavioural testing to suggest that chronic peripheral motor impairment is associated with structural and functional differences in brain regions involved in movement production and/or the perception of visual motion and body form. Specifically, we observed a clear pattern of concordant grey matter, white matter and functional connectivity differences in patients experiencing loss of function and pain as a result of massive irreparable rotator cuff tear in comparison with unaffected control participants.

The observed brain differences extended beyond the cortical regions traditionally associated with chronic pain (S1) to include cortical regions involved in reach and grasp (AIP/IPL) and body/motion perception (MT/TPJ). Of particular note was the concurrence of the results observed across multiple independent imaging modalities (Fig. 5): the observed cluster of lower FA of patients’ right ILF (diffusion‐weighted MRI; Fig. 4) was directly adjacent to the observed clusters of reduced functional connectivity in right MT (resting‐state fMRI; Fig. 3) and reduced grey matter density in left TPJa (T1‐weighted MRI; Fig. 2). In addition, the unexpected structural and functional differences in higher order visual areas observed in Study A were supported by follow‐up behavioural testing in Study B, which offered initial evidence for differences in the perception of visual motion and body form in cuff tear patients compared with control participants.

**Figure 6:**
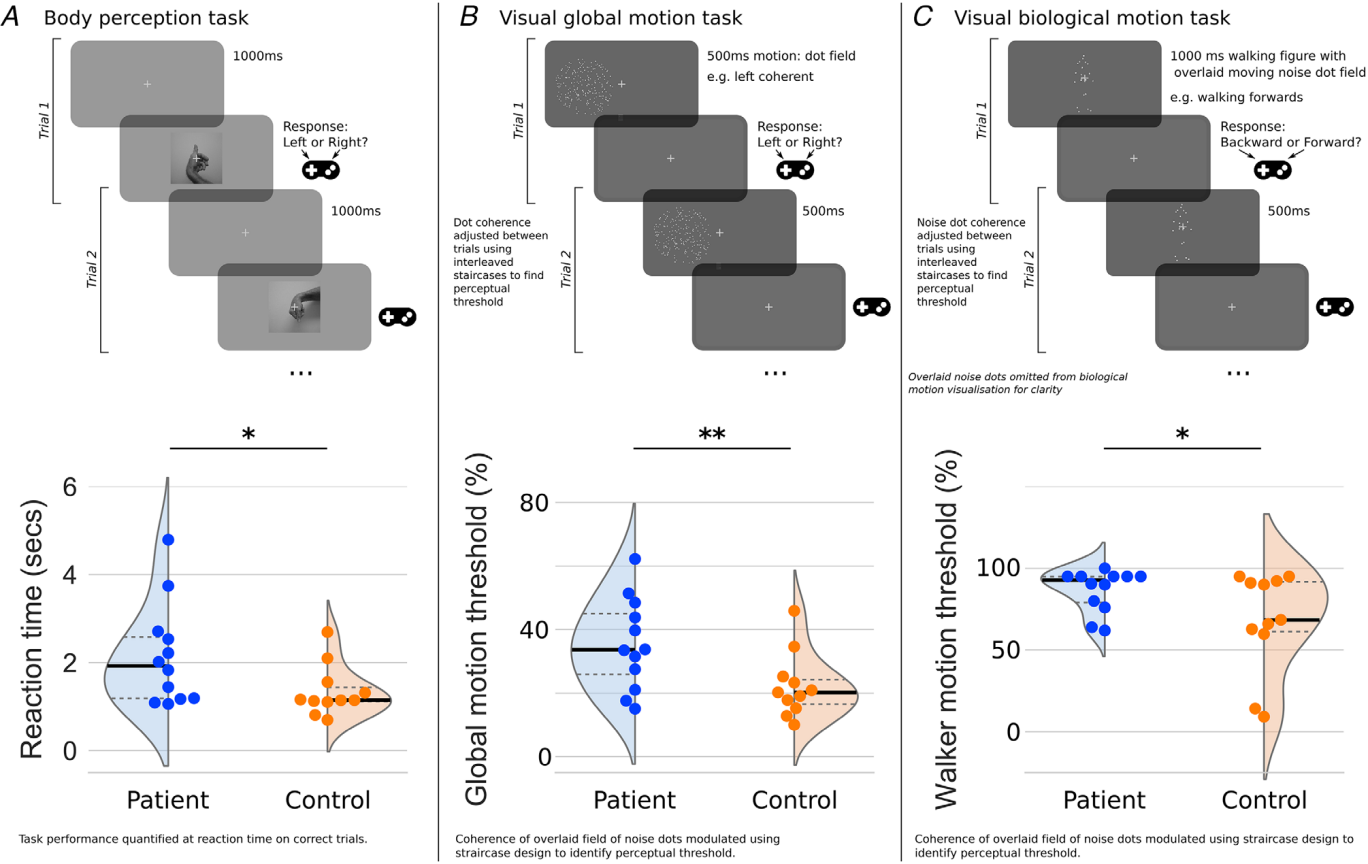
Massive irreparable rotator cuff tear patients exhibit poorer performance in tasks of body and motion perception compared with unaffected control participants A cohort independent of the brain imaging data set show evidence of diminished perceptual abilities in tasks associated with the cortical regions identified from the brain imaging data presented in Figs 2–4. Cuff tear patients were significantly slower in their judgement of hand laterality in a body perception task (*A*). Cuff tear patients also showed significantly higher perceptual thresholds (i.e. poorer performance) in tasks of visual global motion perception (*B*) and visual biological motion perception (*C*) compared with unaffected controls. Cuff tear patients did not differ from the control group in low level visual perception of contrast or acuity. [Color figure can be viewed at wileyonlinelibrary.com]

### Cuff tear patients show reduced grey matter density in the somatosensory cortex

Patients with cuff tear exhibited reduced grey matter integrity (FA) in the somatosensory cortex compared with unaffected controls (Fig. 2 *A*). The region of lower grey matter density in cuff tear patients in Brodmann area 2 of S1 was adjacent to the anatomical hand knob (Makin & Flor, 2020). A comparison of this S1 region to reference functional maps of the cortex revealed the region to be directly adjacent, slightly caudal, and inferior to the representations of the individual fingers in the somatosensory cortex (Fogassi & Luppino, 2005).

The lower grey matter density observed in cuff tear patients is consistent with studies of a number of chronic pain conditions (Maman *et al*. 2009; Makin *et al*. 2013; Mao *et al*. 2013), including phantom pain after upper limb amputation (Mountcastle, 2005). Some amputee literature suggests that the loss of grey matter density in S1 is associated with lower levels of ongoing pain. The amputee literature posits that this is because chronic nociceptive inputs maintain the structural integrity of the cortex, which plays into changes in long‐range brain networks associated with chronic pain (Mountcastle, 2005; Neri *et al*. 2009; Newbold *et al*. 2020). Further investigation in larger cohorts is required to assess whether this correlation between pain and cortical structure generalises to musculoskeletal disease.

### Cuff tear is associated with lower grey matter density in reach and grasp regions of the cortex, and this is mirrored in deficits of body/action perception

Lower grey matter density was observed in regions of the left anterior intra‐parietal cortex (AIP) and more generally in the inferior parietal lobule (IPL), identified as the PF complex by the Glasser atlas (Fig. 2). The region of the PF complex with relatively lower grey matter density in cuff tear patients is directly caudal to S1, traditionally classified as rostral Brodmann area 40. The same region has been directly implicated in the motor control of the shoulder in patients with instability in the joint (MNI coordinates of fMRI activation: −56, −36, 44) (Nichols *et al*. 2005). More generally, these brain regions are implicated in the encoding of motor information during action observation (Nickerson *et al*. 2017). AIP is specifically implicated in grasp formation and intended goal‐dependent reach‐to‐grasp manipulative actions (Oreja‐Guevara *et al*. 2004; O'Neill *et al*. 2020), including a role in the observation of these movements (Peirce, 2007, 2009).

IPL has been implicated in motor control (Pelletier *et al*. 2015). In meta‐analyses, the left IPL in particular has been consistently implicated in motor imagery related to the upper limb (Nickerson *et al*. 2017), as well as action recognition, understanding and imitation (Pelphrey *et al*. 2003, 2004; Pelletier *et al*. 2015). Previous data suggest that judgements of hand laterality engage brain networks including IPL (Piitulainen *et al*. 2012). The observed structural differences in AIP/IPL in cuff tear patients (Fig. 2) concur with the relative deficits in hand laterality judgements we report in an independent cohort of cuff tear patients (Fig. 6). The left pulvinar also exhibited lower grey matter density in the cuff tear group. Studies in macaques have shown this thalamic region to be implicated in manual target selection and limb use (Rizzolatti & Craighero, 2004; Reinersmann *et al*. 2010; Roy *et al*. 2017).

### Massive irreparable rotator cuff tear is associated with relative deficits in visual motion and body form perception: evidence from multimodal neuroimaging and behavioural data

Cuff tear patients showed a cluster of structural and functional alterations in and around the temporal–parietal junction (TPJ), including lower grey matter density and lower cortical thickness in the anterior TPJ itself (Fig. 2), lower FA in underlying white matter (Fig. 4), and differences in the resting‐state motor network, which showed reduced connectivity to the nearby MT cortex (Fig. 3). The observed differences in TPJ motivated us to explore deficits in body form/shape perception in an independent patient group. Similarly, the observed differences in MT motivated us to explore patient deficits in the perception of motion, both global and biological.

The TPJ integrates multisensory body information (Saxe *et al*. 2004; Saranathan *et al*. 2020), amongst a wide variety of other functions related to self‐perception. It has been implicated in the mental imagery of one's own body or imagined body transformations (Schmidt‐Wilcke *et al*. 2006; Scholz *et al*. 2009; Seminowicz *et al*. 2011; Schumaier *et al*. 2020). Cortical regions where we observed lower grey matter density and reduced cortical thickness around the posterior superior temporal sulcus and anterior TPJ respond strongly to biological human motion (Sher *et al*. 1995; Smith *et al*. 2006; Smith & Nichols, 2009).

Our dual regression analysis of the resting‐state fMRI highlighted differences in the activity of a region of visual cortex, called MT, in cuff tear patients. Specifically, this region showed reduced functional connectivity to the resting‐state motor network. Importantly, the specific difference in functional connectivity observed in the resting‐state motor network was not associated with a global difference between patients and controls in the overall strength of the motor network, which suggests a degree of reorganisation within the network rather than a general reduction in its activity in the patient group (Fig. 3 *B*).

Although MT is not normally considered a part of the resting state motor network, in this case the two groups under study showed very different connectivity to this region. Cuff tear patients showed low correlation or anticorrelation between activity in the motor RSN and MT, while the control group showed positive correlation between activity in the motor RSN and MT (Fig. 3). The Neurosynth meta‐analysis of the MT cluster highlighted a very clear and well‐established role for this region in motion and action perception (Fig. 3 *E*). MT does not specifically detect visual motion or undertake any complex processing of the fundamental motion signal: primary visual cortex subserves this function. The role of MT appears to be in integrating motion signals across space in order to capture patterns of motion over wider areas of the visual field (Smith, 2002). Evidence from primate and human studies suggests that V5/MT is engaged during the production of continuous visually tracked movements, but not ballistic movements (Tunik *et al*. 2007; Tsao *et al*. 2010; Tracey, 2016); we therefore speculate that the functional de‐coupling of motor cortex and MT in the patient group may be a consequence of long term disuse of the affected upper limb for such target driven skilled movements.

In light of the convergent neuroimaging evidence of reduced integrity in regions responsible for body and motion perception, we investigated further with behavioural experiments in an independent cohort of massive irreparable rotator cuff tear patients and control participants (Study B). We applied well‐established tasks to quantify body perception (hand laterality judgement), biological motion perception (point‐light walking figure) and global motion (random dot kinematograms).

These tests concurred with the imaging results, further supporting our hypothesis that the musculoskeletal impairment caused by cuff tear is associated with deficits that affect the perception of body form and motion. Patients showed evidence that suggested they had deficits in the perception of biological motion, global motion and the perception of body form relative to control participants in our sample (Fig. 6).

Importantly, we found no evidence of generalised deficits in visual acuity, contrast acuity or reaction time in response to a fixed cue in the visual motion tasks, suggesting some degree of specificity in the deficits we observed in the patient group. Furthermore, the presence of relatively easy ‘catch’ trials in the visual motion tasks allowed us to exclude data from participants who were unable to sufficiently attend to or focus on the task. Moreover, during recruitment, participants were screened for any obvious cognitive or documented neurological deficits.

Notably, our imaging and behavioural data were acquired in two separate and independent cohorts, allowing us to be more confident in the generalizability of our results. Specifically, we avoided the pitfalls of circular inference that would occur by undertaking the behavioural testing and imaging in the same cohort; here the imaging data only informed the behavioural tests we applied in a different group (Tunik *et al*. 2005).

Nonetheless, the behavioural testing presented here represents an exploratory follow‐up experiment informed by our neuroimaging data; it is not an exhaustive sample. Given the age of the populations under study, a variety of additional factors, including subtle differences in attention, memory or general cognition, cannot be discounted as underlying our observed results. However, we posit that the complementarity of our results across imaging and behavioural testing in two independent cohorts represents an encouraging first step in investigating such visual deficits in musculoskeletal disorders.

Deficits in hand laterality judgements have previously been well reported in chronic pain conditions such as complex regional pain syndrome (Vanrie & Verfaillie, 2004), phantom pain (Kim *et al*. 2018) and focal dystonia (Wani *et al*. 2016). Deficits in visual motion perception, however, have not previously been associated with musculoskeletal disorders. Studies using the same point‐light walker stimuli employed in the present study have reported deficits in the perception of biological motion in movement disorders such as paraplegia and Parkinson's disease (Yamaguchi *et al*. 2001; Wilke *et al*. 2010).

### How do peripheral and central changes interact in musculoskeletal disease?

The notion that the brain is involved in musculoskeletal disease has been discussed for some time; indeed, it is in keeping with our understanding of plasticity as a cyclical interplay between bottom‐up and top‐down influences (Acuña *et al*. 1983; Beckmann *et al*. 2005; Bach, 2007). The data presented in this study support this notion indirectly without demonstrating causality: there appear to be differences in the motor regions of cuff tear patients compared with control participants, and we hypothesise that these differences were caused by a cyclical process of reduced motor efferent and somatosensory afferent activity. The combination of pain and loss of function brought about by cuff tear results in reduced use of the affected limb. From studies involving experimental arm casting, we know that disuse of a limb can functionally disconnect the brain regions responsible from other parts of the wider motor network (Arzy *et al*. 2006). In this case, it is challenging to ascertain whether a lack of efferent or afferent activity is the initial driving force.

A novel observation in this study is that the observed differences in brain structure and function extend into regions distinct from the traditional motor network, notably areas responsible for the perception of visual motion and body form. This observation is supported by evidence from both the imaging and the behavioural experiments, although further confirmation in other musculoskeletal disorders is necessary to assess generalizability. Massive irreparable rotator cuff tear is associated with chronic motor impairment over a course of years. As such, we speculate that the resulting long‐term alterations in the afferent and efferent activity of the motor network go on to induce changes in the activity of other related cortical networks, such as the parietal and temporal mirror neuron system (Yousry *et al*. 1997; Yarkoni *et al*. 2011; Zapparoli *et al*. 2014), resulting in the structural and functional differences that we observe in our patient cohort.

A further fundamental question remains: are these brain changes merely an epiphenomenon, reversed by peripheral interventions and the restoration of afferent/efferent activity, or do they represent a lasting change and therefore a potential target for adjunct therapies? For musculoskeletal conditions in which effective peripheral interventions are available, for example after a hip replacement, there is limited evidence of the reversal of these maladaptive changes or musculoskeletal training (Bach, 1996; Zatorre *et al*. 2012). However, studies of pain have taught us that chronic conditions are not necessarily a mere extension of acute symptoms, but rather that they represent a distinct disease process with a central aetiology (Zimmerman, 2004). In massive irreparable cuff tear and other musculoskeletal conditions that lack disease modifying treatments or surgical repair, an understanding of longitudinal changes in the motor system as the disease progresses will be vital to understanding the role of the brain and any potential new targets for therapy or rehabilitation.

## Additional information

### Competing interests

The authors declare no competing interests.

### Author contributions

V.C., R.A., D.N., R.S., I.T. and J.K. conceived and planned the experiments. V.C., B.D., R.S., S.L., I.T. and J.K. provided technical input on data acquisition and analysis. V.C., C.E., R.A., D.N., C.B., B.D., S.K., R.S., S.L. and J.K. acquired the data. V.C., C.E., C.B., B.D., S.L., I.T. and J.K. conducted the data analysis. V.C., C.E., R.A., D.N., C.B., B.D., S.K., R.S., S.L., I.T. and J.K. contributed to the interpretation of the results. J.K. took the lead in writing the manuscript. All authors offered critical feedback and helped refine the analysis and the manuscript. All authors have read and approved the final version of this manuscript and agree to be accountable for all aspects of the work in ensuring that questions related to the accuracy or integrity of any part of the work are appropriately investigated and resolved. All persons designated as authors qualify for authorship, and all those who qualify for authorship are listed.

### Funding

This work was supported by a grant from the Torbay Medical Research Fund. J.K. is supported by a Sir Henry Wellcome Postdoctoral Fellowship (204696/Z/16/Z). S.K. is supported by a Wellcome Trust Strategic Award (104943/Z/14/Z).

## Supporting information


Statistical Summary Document

Neurosynth data for motor resting state network dual regression analysis: control>patient

Neurosynth data for voxel‐based morphometry clusters: control>patient

## Data Availability

Group mean and statistical maps from neuroimaging data and behavioural data files are available from the corresponding author upon reasonable request. Participants did not consent to open sharing of raw MRI data.
